# Supplementary material for: Designing stable neural networks using convex analysis and ODEs
Source: Physica D. Author manuscript; Available in PMC 2026 Oct 16. (PMC7619584; doi:10.1016/j.physd.2024.134159)
Supplement: Supplementary file 1 [file EMS218485-supplement-Appendix.pdf]

Appendix. Comparisons of computational costs

In this section, we will give estimates of relevant timings and memory measurements for the methods that we compared in Section 3. In each instance, we perform these measurements on a single minibatch, with the same settings as were used for training, so that the timings give a reasonable view of the cost of training.

To perform the timing and memory measurements, we use functionality provided by PyTorch for NVIDIA GPUs. Replacing the comments in Code Snippet 1 by appropriate code to set up the model and the code to be measured, we get code that can be used for these measurements.

Code Snippet 1: GPU timing and memory measurement code

```
import torch

""" code to set up model on device """

start = torch.cuda.Event(enable_timing=True)
end = torch.cuda.Event(enable_timing=True)
torch.cuda.reset_max_memory_allocated(device)

start.record()
""" run code involving model """
end.record()
torch.cuda.synchronize(device)

time = start.elapsed_time(end)
memory = torch.cuda.max_memory_allocated(device)
```

In the tables that follow, t_{FB} is the time taken to perform a forward propagation and backpropagation, t_P is the time taken to perform an iteration of the power method, t_F is the time taken to perform a forward propagation and we refer to the GPU memory allocated by mem.

A.1. Adversarial robustness examples

As in the examples studied in Section 3.2, we perform measurements on a single minibatch consisting of 512 images from CIFAR-10. The proposed network initially performs 15 integration steps (corresponding to $\text{NonExpNet}_{\text{init}}$ in Table A.1). During training with the adaptive approach, the maximum number of integration steps taken by the proposed network is 63 (corresponding to $\text{NonExpNet}_{\text{max}}$ in Table A.1).

A.2. Denoising examples

As in the examples studied in Section 3.3, we perform measurements on a single minibatch consisting of 5 images from BSDS500. All of the proposed networks initially perform 10 integration steps (corresponding to $\text{Euler}_{\text{init}}$, $\text{Heun}_{\text{init}}$ and RK4_{init} in Table A.2). During training with the adaptive approach, the maximum number of integration steps taken by the proposed networks are 23, 22 and 26 steps for the networks using the Euler, Heun and RK4 integrator, respectively (shown in Table A.2 as $\text{Euler}_{\text{max}}$, Heun_{max}, RK4_{max}).

Table A.1
Timings and memory measurements for the models compared in Section 3.2.

Method	Training			Inference	
	t_{FB} (ms)	t_P (ms)	mem (MB)	t_F (ms)	mem (MB)
ResNet	20	N/A	1,758	6	896
ResNet-AT	163	N/A	1,782	6	896
$\text{NonExpNet}_{\text{init}}$	20	5	1,758	6	896
$\text{NonExpNet}_{\text{max}}$	83	5	4,782	26	931

Table A.2
Timings and memory measurements for the models compared in Section 3.3.

Method	Training			Inference	
	t_{FB} (ms)	t_P (ms)	mem (MB)	t_F (ms)	mem (MB)
TV	N/A	N/A	N/A	1,447	1,086
DnCNN	143	N/A	8,563	45	1,519
$\text{Euler}_{\text{init}}$	102	8	8,086	37	2,293
$\text{Euler}_{\text{max}}$	236	8	15,612	84	2,485
$\text{Heun}_{\text{init}}$	212	8	14,068	77	2,679
Heun_{max}	466	8	27,964	168	2,870
RK4_{init}	443	8	26,034	163	3,065
RK4_{max}	1,155	8	63,090	423	3,450
